# Integrating phylogenies into single-cell RNA sequencing analysis allows comparisons across species, genes, and cells

**DOI:** 10.1371/journal.pbio.3002633

**Published:** 2024-05-24

**Authors:** Samuel H. Church, Jasmine L. Mah, Casey W. Dunn

**Affiliations:** Department of Ecology and Evolutionary Biology, Yale University, New Haven, Connecticut, United States of America

## Abstract

Comparisons of single-cell RNA sequencing (scRNA-seq) data across species can reveal links between cellular gene expression and the evolution of cell functions, features, and phenotypes. These comparisons evoke evolutionary histories, as depicted by phylogenetic trees, that define relationships between species, genes, and cells. This Essay considers each of these in turn, laying out challenges and solutions derived from a phylogenetic comparative approach and relating these solutions to previously proposed methods for the pairwise alignment of cellular dimensional maps. This Essay contends that species trees, gene trees, cell phylogenies, and cell lineages can all be reconciled as descriptions of the same concept—the tree of cellular life. By integrating phylogenetic approaches into scRNA-seq analyses, challenges for building informed comparisons across species can be overcome, and hypotheses about gene and cell evolution can be robustly tested.

## Abstract 2

Comparisons of single-cell RNA sequencing (scRNA-seq) data across species can reveal links between cellular gene expression and the evolution of cell functions, features, and phenotypes. This Essay contends that, by integrating phylogenetic approaches into scRNA-seq analyses, hypotheses about gene and cell evolution can be robustly tested.

## Introduction

Single-cell RNA sequencing (scRNA-seq) generates high-dimensional gene expression data from thousands of cells from an organ, tissue, or body [1]. Single-cell expression data are increasingly common, with new animal cell atlases being released every year [2–6]. The next steps will be to compare such atlases across species [2], identifying the dimensions in which these results differ and associating these differences with other features of interest [7]. Because all cross-species comparisons are inherently evolutionary comparisons, such analyses present an opportunity to integrate approaches from the field of evolutionary biology, and especially phylogenetic biology [8]. Drawing concepts, models, and methods from these fields will help to overcome central challenges with comparative scRNA-seq analysis, especially in how to draw coherent comparisons over thousands of genes and cells across species. In addition, this synthesis of concepts will help avoid the unnecessary reinvention of analytical methods that have already been rigorously tested in evolutionary biology for other types of data, such as morphological and molecular data.

Comparative gene expression analysis has been used for decades to answer evolutionary questions such as how changes in gene expression are associated with the evolution of novel functions and phenotypes [9]. The introduction of scRNA-seq technology has led to a massive increase in the scale of these experiments [1], from working with a few genes or a few tissues, to assays that cover the entire transcriptome, across thousands of cells in a dissociation experiment. Comparative scRNA-seq analysis therefore enables evolutionary questions to be scaled up, for example: how has the genetic basis of differentiation evolved across cell populations and over time; what kinds of cells and gene expression patterns were likely present in the most recent common ancestor; what changes in cell transcriptomes are associated with the evolution of new ecologies, life-histories, or other features; how much variation in cellular gene expression do we observe over evolutionary time; which changes in gene expression are significant (i.e., larger or smaller than we expect by chance); which genes show patterns of correlated expression evolution; and can evolutionary screens detect novel interactions between genes?

**Fig 1:**
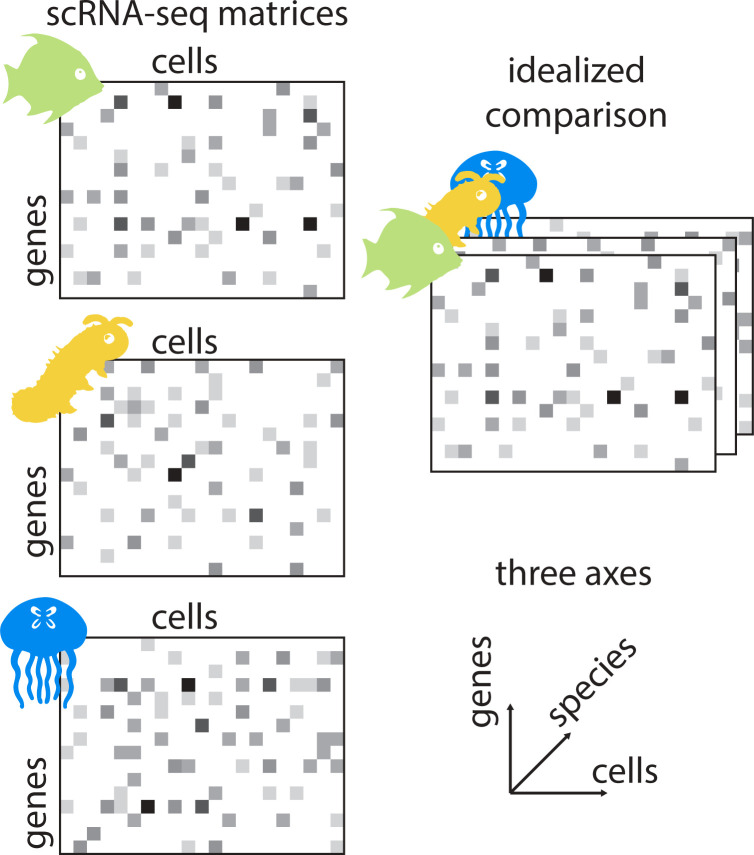
Comparing count matrices across species. scRNA-seq experiments generate count matrices, shown here with columns as cells and rows as genes. Higher expression counts for a given gene in a given cell are depicted with darker shading. In an idealized comparison, count matrices across species would be aligned to form a 3D tensor of expression across cells, genes, and species. In reality, there is no expectation of one-to-one correspondence or independence for any of the 3 axes. Instead, relationships between species, genes, and cells are described by their respective evolutionary histories, as depicted with phylogenies.

In comparative scRNA-seq studies, the results of individual experiments are analyzed across species. These scRNA-seq experiments usually generate matrices of count data with measurements along 2 axes: cells and genes (Fig 1). Comparative scRNA-seq analysis adds a third axis: species. At first glance, it might make sense to try and align scRNA-seq matrices across species, thereby creating a 3D tensor of cellular gene expression. But neither genes nor cells are expected to share a one-to-one correspondence across species. In the case of genes, gene duplication (leading to paralogous relationships) and gene loss are rampant [10]. In the case of cells, there is rarely justification for equating 2 individual cells across species; instead, populations of cells (“cell types”) are typically compared [11]. Therefore to align matrices, an appropriate system of grouping both dimensions must first be found. This is essentially a question of homology [12]: which genes and cell types are homologous, based on their relationship to predicted genes and cell types in the common ancestor.

**Fig 2:**
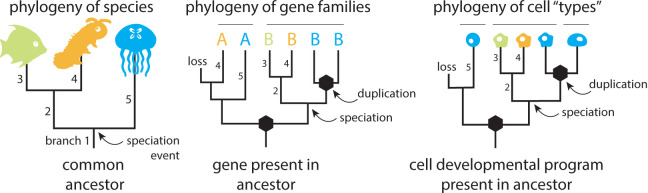
Phylogenies of species, genes, and cells. Species phylogenies contain speciation events as nodes in a bifurcating tree. Gene phylogenies contain both gene duplication events (black hexagons) and speciation events (unmarked) at nodes. Cell phylogenies also include both speciation and duplication events; here, duplication events represent a split in the program of cellular development that leads to differentiated cell types [13]. Branches from the species phylogeny (numbered branches) can be found within gene and cell phylogenies. Note that gene families are strictly defined by ancestry, but cell types have historically been defined by form, function, or patterns of gene expression [15]. This means that groups of cells identified as the same “type” across species may reflect paraphyletic groups [11], as depicted in the second cell type in this tree.

Questions about homology can be answered using phylogenies [12]. Species relationships are defined by their shared ancestry, as depicted using a phylogeny of speciation events (Fig 2). Gene homology is also defined by shared ancestry, depicted using gene trees that contain nodes corresponding to either speciation and gene duplication events. Cell homology inference requires assessing the evolutionary relationships between cell types [12,13], defined here as populations of cells related via the process of cellular differentiation and distinguishable from one another (e.g., by using molecular markers) [14]. Relationships between cell types can be represented with cell phylogenies that, like gene trees, contain both speciation and duplication nodes [13]. As with genes, the evolutionary relationships between cell types may be complex, as differentiation trajectories drift, split, or are lost over evolutionary time [7,13,15].

In this Essay, we illustrate a tree-based framework for comparing scRNA-seq data and contrast this framework with existing methods. We describe how we can use trees to identify homologous and comparable groups of genes and cells, based on their predicted relationship to genes and cells present in the common ancestor. We advocate for mapping data to branches of phylogenetic trees to test hypotheses about the evolution of cellular gene expression, describing the kinds of data that can be compared and the types of questions that each comparison has the potential to address. Finally, we reconcile species phylogenies, gene phylogenies, cell phylogenies, and cell lineages as different representations of the same concept—the tree of cellular life.

## Comparisons across species

Shared ancestry between species will impact the results of all cross-species analyses and should therefore influence expectations and interpretations [16]. For scRNA-seq data, this has several implications. First, species are expected to be different from one another, given that they have continued evolving since diverging from their common ancestor. Therefore, by default, many differences in cellular gene expression are expected across the thousands of measurements in an scRNA-seq dataset. Second, the degree of difference is expected to correlate with time since the last common ancestor. The null expectation is that closely related species will have more cell types in common, and that those cells will have more similar patterns of gene expression than more distantly related species. The structure of this similarity can be approximated with a species phylogeny calibrated to time.

Methods for the evolutionary comparison of scRNA-seq data have already been proposed in packages such as SAMap [7]. These packages have overcome significant challenges, such as how to account for non-orthologous genes (see the section Comparisons across genes). However, up to now these methods have relied on pairwise comparisons of species, rather than phylogenetic relationships. The problems with pairwise comparisons have been well-described elsewhere [17]; briefly, they result in pseudo-replication of evolutionary events. This pseudo-replication is of increasing concern as comparisons are drawn across a greater number of taxa and across more closely related species. By contrast, an evolutionary comparative approach maps evolutionary changes to branches in the phylogeny [8,10]. With this approach, data are assigned to the tips of a tree, and ancestral states are reconstructed using an evolutionary model. Evolutionary changes are then calculated as differences between ancestral and descendant states, and the distribution of evolutionary changes along branches are analyzed and compared [18].

Shifting toward a phylogenetic approach to comparative scRNA-seq analysis unlocks new avenues of discovery, including tests of coevolution of cellular gene expression and other features of interest [9], as well as evolutionary screens for signatures of correlated gene and cell modules [19]. In phylogenetic analyses, statistical power depends on the number of independent evolutionary events rather than on the absolute number of taxa [8]. Therefore, the choice of which species to compare is critical, especially when comparisons can be constructed to capture potential convergence.

One consideration when comparing species is the degree to which the history of scientific study has favored certain organisms (e.g., model organisms) [20]. This is especially relevant to single-cell comparisons, as more information about cell and gene function is available for some species (e.g., mice and humans) than for others. This creates a risk of bias toward observing described biological phenomena, while missing the hidden biology in less well-studied organisms [20]. Consider the identification of “novel” cell types based on the absence of canonical marker genes: because most canonical marker genes were originally described in well-studied species, cell type definitions that rely on these will necessarily be less useful in the context of other species [2].

Technologies such as scRNA-seq have great potential to democratize the types of data collected [2]. For example, scRNA-seq allows all genes and thousands of cells to be assayed, rather than a curated list of candidates. To leverage this to full effect, researchers need to acknowledge the filtering steps in their analyses, including how orthologous gene sequences are identified and how cell types are labeled.

## Comparisons across genes

Due to gene duplication and loss, there is usually not a one-to-one correspondence between genes across species [21]. Instead, evolutionary histories of genes are depicted using gene trees (Fig 2). Pairs of tips in gene trees may be labeled as “orthologs” or “paralogs,” based on whether they descend from a node corresponding to a speciation or gene duplication event [22]. Gene duplication happens both at the individual gene level and in bulk, via whole or partial genome duplication [21]. Gene loss means that comparative scRNA-seq matrices may be sparse, not only due to a failure to detect a gene, but also because genes in one species often do not exist in another.

The authors of many cross-species comparisons have confronted the challenge of finding equivalent genes across species [23], and often start by restricting analyses to sets of one-to-one orthologs [24]. However, there are several problems with this approach [22]: one-to-one orthologs are only well-described for a small set of very well-annotated genomes [23]; the number of one-to-one orthologs decreases rapidly as species are added to the comparison, and as comparisons are made across deeper evolutionary distances [7]; and the subset of genes that can be described by one-to-one orthologs is not randomly drawn from across the genome, they are enriched for indispensable genes under single-copy control [25]. New tools like SAMap are expanding the analytical approach beyond one-to-one orthologs to the set of all homologs across species [7]. Homolog groups are identified with a clustering algorithm, by which genes are separated into groups with strong sequence or expression similarity. These may include more than 1 representative gene per species. Gene trees can then be inferred for these gene families, and duplication events mapped to individual nodes in the gene tree.

But how can cellular expression measures be compared across groups of homologous genes? One option is to use summary statistics, such as the sum or average expression per species for genes within a homology group [26]. However, these statistics might obscure or average over real biological variation in expression that arose subsequent to a duplication event (among paralogs) [19]. An alternative approach is to connect genes via a similarity matrix, and then make all-by-all comparisons that are weighted on the basis of putative homology [7]. A third approach is to reconstruct changes in cellular expression along gene trees, rather than along the species tree [10,27]. Here, evolutionary changes are associated with branches descending from either speciation or duplication events. Such an approach has been demonstrated for bulk RNA sequencing, in which gene trees were inferred from gene sequence data and cellular expression data were assigned to tips of a gene tree. In this approach, ancestral states and evolutionary changes are calculated and equivalent branches between trees are identified using “species branch filtering” [27]. Branches between speciation events can be unambiguously equated across trees based on the composition of their descendant tips (see numbered branches in Fig 2) and changes across equivalent branches of a cell tree analyzed (e.g., to identify significant changes, signatures of correlation).

Mapping cellular gene expression data to branches of a gene tree sidesteps the problem of finding sets of orthologs by incorporating the history of gene duplication and loss into the analytical framework. One technical limitation is that the ability to accurately reconstruct gene trees depends on the phylogenetic signal of gene sequences, which in turn depends on the length of the gene, the mutation rate, and the evolutionary distance in question [28]. These dynamics are such that, for some genes, it may not be possible to robustly reconstruct the topology, although targeted taxon sampling can improve gene tree inference across a wider range of histories.

## Comparisons across cells

As with genes, there is usually not an expectation of a one-to-one correspondence between cells across species. Individual cells can rarely be equated, with notable exceptions such as the zygote or the cells of certain eutelic species (which have a fixed number of cells). Instead, the homology of groups of cells (cell types) are usually considered, with the hypothesis being that the cell developmental programs that give rise to these groups are derived from a program present in the shared ancestor [11].

Similarly, a one-to-one correspondence between cell types across species is also not expected, as cell types may be gained or lost over evolutionary time. The relationships between cell types across species can be described using phylogenetic trees. These cell phylogenies are distinct from cell lineages (the bifurcating trees that describe cellular divisions within an individual developmental history). Nodes in cell lineages represent cell divisions, whereas nodes in cell phylogenies represent either speciation events or splits in differentiation programs that lead to novel cell types (Fig 2). The evolutionary histories of cell types may not follow a strict bifurcating pattern of evolution, as elements of differentiation programs are mixed and combined. However, evidence from inference on sequence data shows that the majority of relationships between cell types can be represented as trees [15].

The term “cell type” has been used for several distinct concepts [15], including cells that are defined and distinguished by their position in a tissue, their form, function, or in the case of scRNA-seq data, their relative expression profiles, which fall into distinct clusters [14]. Homology of structures across species is often inferred using many of the same criteria: position, form, function, and gene expression patterns [29]. The fact that the same principles are used for inferring cell types and cell homologies presents both an opportunity and obstacles for comparative scRNA-seq analysis. The same methods that are used for identifying clusters of cells within species can potentially be leveraged to identify clusters of cells across species. This could be done simultaneously, inferring a joint cell atlas in a shared expression space [7], or it could be done individually for each species and subsequently merged [2,23]. In either case, this inference requires contending with the evolutionary histories between genes and species, described above.

One obstacle is that, because cell types are not typically defined according to evolutionary relationships [15], cells labeled as the same type across species may constitute paraphyletic groups [11]. A solution to this problem is to use methods for reconstructing evolutionary relationships to infer the cell tree [15,30] (Fig 2). This method is distinct from an approach in which cell types are organized into a taxonomy on the basis of morphological or functional similarity [14]; instead, this approach uses an evolutionary model to infer the evolutionary history, including potential duplication and loss. It has the additional advantage of generating a tree, comparable to a species or gene tree, onto which cellular characters can be mapped and their evolution described [15]. Methods for inferring cell trees from expression data have been described in detail elsewhere [31–33]. Using this approach, cell trees are inferred (e.g., using expression of orthologous genes as characters in an evolutionary model) and gene expression data are assigned to the tips of the cell tree. Ancestral states and evolutionary changes are then calculated and changes along branches are analyzed (e.g., to identify changes in gene expression associated with the evolution of novel cell types).

As with genes, the ability to infer cell trees depends on the phylogenetic signal of cellular traits such as cellular gene expression profiles. Although the phylogenetic signal of expression data has been demonstrated in various contexts [32–35], certain cell types, such as cancer cells that follow a distinct mode of evolution, may exhibit less tree-like structures [34]. Species-specific effects and signals from correlated evolution may also obscure cell phylogenetic signals. Given the low-rank nature of cell gene expression, dimensional reduction techniques such as principal component analysis have been employed to extract and clarify phylogenetic signals [33]. Other complexities, such as naturally occurring instances of cellular reprogramming or transdifferentiation could also potentially obscure phylogenetic signals, although cellular identity is thought to be stable under most circumstances [36].

Another obstacle to comparing single-cell datasets are reported batch effects [26] across experiments, which may need to be accounted for via integration [23]. When considering these effects, it is critical to remember that the null expectation is that species are different from one another. Naive batch integration practices have no method for distinguishing technical effects from the real biological differences that are the target of study in comparative scRNA-seq analysis [23]. Other approaches (e.g., LIGER [37] or Seurat [38]) are reportedly able to distinguish and characterize species-specific differences [23]. Given that null hypotheses are still being developed [16] for how much variation in expression is expected to be observed across species [19], we hold that cross-species integration should be treated with caution until elucidation of the approach can robustly target and strictly remove technical batch effects.

A final obstacle is that cell identities and homologies may be more complex than can accurately be captured by categorization into discrete clusters or cell types, particularly when considering multiple cell states along a differentiation trajectory [14,15]. Single-cell experiments that include both progenitor and differentiated cells can reveal the limits of clustering algorithms [39]. In these experiments, there may or may not be obvious boundaries for distinguishing cell states. In cases where boundaries are arbitrary, the number of clusters, and therefore the abundance of cells within a cluster will depend on technical and not biological inputs, such as the resolution parameter that the user predetermines for the clustering algorithm. A solution here is to define homology for the differentiation trajectory, rather than for individual clusters of cells [26]. This can be accomplished by defining anchor points where trajectories overlap in the expression of homologous genes while allowing for trajectories to have drifted or split over evolutionary time, such that sections of the trajectories no longer overlap [15]. Cellular homologies within a trajectory may be more difficult to infer, as this requires contending with potential heterochronic changes to differentiation (e.g., as cell differentiation evolves, genes may be expressed relatively earlier or later in the process) [26].

## Constructing comparisons of scRNA-seq data

**Fig 3:**
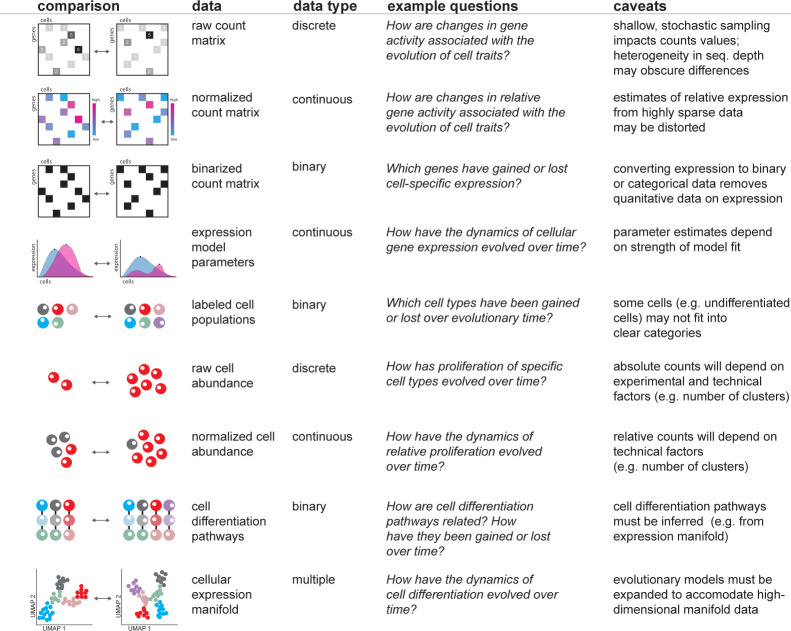
Types of scRNA-seq data that can be mapped onto a phylogeny. Several types of scRNA-seq data could potentially be mapped onto a phylogeny. Nine types of data are shown, along with example questions that can be addressed and caveats to be considered.

Single-cell comparisons potentially draw on a broad range of phylogenetic comparative methods for different data types, including binary, discrete, continuous, and categorical data [40] (Fig 3). The primary data structure of scRNA-seq is a matrix of integers, representing counts of transcripts or unique molecular identifiers for a given gene within a given cell [41]. In a typical scRNA-seq analysis, this count matrix is passed through a pipeline of normalization, transformation, dimensional reduction, and clustering [42,43]. The decisions of when during this pipeline to draw a comparison determines the data type, questions that can be addressed, and caveats that must be considered.

### Gene expression data

Unlike bulk RNA sequencing, where counts are typically distributed across a few to dozens of samples, scRNA-seq counts are distributed across thousands of cells. The result is that scRNA-seq count matrices are often shallow and sparse [44]. The vast majority of counts (often >95%) in standard scRNA-seq datasets are either 0, 1, or 2 [41]. These count values, representing the number of unique molecular identifiers that encode unique transcripts in cells, are discrete, low integer numbers, and not continuous measurements. The high dimensionality and sparse nature of single-cell data therefore present a unique challenge when considering cross-species comparisons [2].

In a standard scRNA-seq approach, expression values are analyzed after depth normalization and other transformations. With depth normalization, counts are converted from discrete, absolute measures to continuous, relative ones (although currently available instruments do not actually quantify relative expression). There is a growing concern that this, and other transformations, are inappropriate for the sparse and shallow sequencing data produced by scRNA-seq [45,46]. Further transformations of the data, such as log transformation or variance rescaling, introduce additional distortions that may obscure real biological differences between species.

Alternatively, counts can be compared across species directly, without normalization or transformation [41]. There are 2 potential drawbacks to this approach. First, count values are influenced by stochasticity due to the shallow nature of sequencing, resulting in uncertainty around integer values. Second, cells are not sequenced to a standard depth. Comparing raw counts does not take this heterogeneity into account, although this can be accomplished using a restricted algebra to analyze counts [41]. Another option is to transform count values to a binary or categorical trait [47]; for example, binning counts into “on” and “off” based on a threshold value and then modeling the evolution of these states on a tree. Analyzing expression as a binary or categorical trait eliminates some of the quantitative power of scRNA-seq, but still allows interesting questions about the evolution of expression dynamics within and across cell types to be addressed.

### Models of expression

A promising avenue for scRNA-seq data is using generalized linear models to analyze expression [46,48,49]. These models describe expression as a continuous trait and incorporate the sampling process using a Poisson or other distribution, avoiding normalization and transformation, and returning fitted estimates of relative expression. These estimates can be compared using models that describe continuous trait evolution. One feature of generalized linear models is that they can report uncertainty values for estimates of relative expression, which can then be passed along to phylogenetic methods to assess confidence in the evolutionary conclusions drawn.

### Cell diversity

In a standard scRNA-seq approach, cells are analyzed in a reduced dimensional space and clustered by patterns of gene expression [43]. There are several types of cellular data that can be compared. The evolution of the presence or absence of cell types can be modeled as a binary trait. When cell type labels are unambiguously assigned, this approach can answer questions about when cell types evolved and are lost. Such a comparison is hampered; however, when cells do not fall into discrete categories [14] or when equivalent cell types cannot be identified across species due to substantial divergence in gene expression patterns. An alternative is to model the evolution of cell differentiation pathways as a binary trait on a tree to ask when pathways, rather than cell types, evolved and have been lost. As with other comparative methods, this approach must contend with complex evolutionary histories, including the potential for convergence as pathways independently evolve to generate cell types with similar functions and expression profiles.

Similarly, the abundance of cells of a given type might be compared across species (for example, to ask how dynamics of cell proliferation have evolved). However, the number of cells within a cluster can be influenced by technical features of the experiment such as the total number of clusters identified (often influenced by user-supplied parameters), as well as where cluster boundaries are defined. An alternative is to compare relative cell abundance values, which may account for experimental factors but is still unreliable as it is susceptible to bias from technical aspects of how cells are dissociated and how clusters are determined.

### Cellular manifolds

One area for further development are methods that can model the evolution of the entire cellular expression manifold—the space that defines cell-to-cell similarity and cellular differentiation—on an evolutionary tree. Practically, this might be accomplished by parameterizing the manifold, for example, by calculating measures of manifold shape and structure such as distances between cells in a reduced dimensional space. The evolution of such parameters could be studied by analyzing them as characters on a phylogenetic tree.

Alternatively, we can envision a method in which entire ancestral landscapes of cellular gene expression are reconstructed, and then the way this landscape has been reshaped over evolutionary time is described. Such an approach would require an expansion of existing phylogenetic comparative models to ones that can incorporate many thousands of dimensions. It would also likely require dense taxonomic sampling to build robust reconstructions.

## Future directions and conclusion

**Fig 4:**
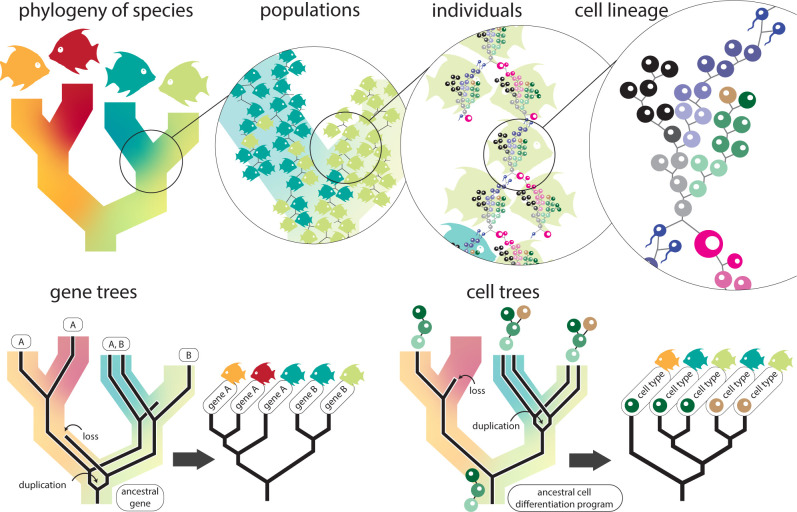
Unification of trees for species, populations, genes, and cells. All cellular life is related by an unbroken chain of cell divisions. Species phylogenies describe the relationship between populations. Populations are themselves a description of the genealogical relationships between individuals. Peering even closer reveals that each individual consists of a lineage of cells, connected to other individuals via reproductive cells. Therefore, species trees, genealogies, and cell lineages are all descriptions of the same concept—the tree of life—but at different scales. Gene trees and cell trees (i.e., cellular phylogenies) describe the evolutionary histories of specific characters within the tree of life. These trees may be discordant with species trees due to duplication, loss, and incomplete sorting in populations.

Comparative scRNA-seq analysis spans the fields of evolutionary, developmental, and cellular biology. Trees depicting relationships across time are the common denominator of these fields. Taking a step back reveals that many of the trees that are typically encountered, such as species phylogenies, gene phylogenies, cell phylogenies, and cell fate maps, can be reconciled as part of a larger whole (Fig 4). Because all cellular life is related via an unbroken chain of cellular divisions, species phylogenies and cell fate maps are 2 representations of the same larger phenomenon, visualized at vastly different scales. Gene trees and cell trees (i.e., cell phylogenies) depict the evolution of specific characters (genes and cells) across populations within a species tree. These characters may have discordant evolutionary histories with each other, and with the overall species phylogeny, due to patterns of gene and cell duplication, loss, and incomplete sorting across populations.

The synthesis of species, gene, and cell trees makes 2 points clear. First, phylogenetic trees are essential for testing hypotheses about cellular gene expression evolution. Mapping single-cell data to trees, whether gene trees, cell trees, or species trees, allows for statistical tests of coevolution, diversification, and convergence. The choice of which trees to use for mapping data will be determined by the questions that need to be answered. For example, mapping cellular expression data to gene trees would allow whether expression evolves differently following gene duplication events (i.e., the ortholog conjecture [50]) to be tested. Second, because the fields of evolutionary, developmental, and cellular biology study the same phenomena at different scales, there is a potential benefit from sharing methods. In the case of scRNA-seq, building evolutionary context around data can prove essential for understanding the fundamental biology, including how to interpret cell types and cellular differentiation trajectories, and how to reconcile gene relationships. An evolutionary perspective is also critical for building robust null expectations of how much variation might be expected to be observed across species [16], which will allow the significance of results to be interpreted as new species atlases come to light. Methods that infer and incorporate trees are essential not only for evolutionary biology, but also for developmental and cellular biology as well. As single-cell data become increasingly available, rather than reinvent methods for building cell trees or comparing across cellular network diagrams, we can draw approaches from the extensive and robust fields of phylogenetic inference and phylogenetic comparative methods. These approaches include Bayesian and Maximum Likelihood inference of trees, evolutionary models, ancestral state reconstruction, character state matrices, and phylogenetic hypothesis testing, among many others [51–53].

Biology has benefited in the past from the synthesis of disparate fields of study, including the modern synthesis of Darwinian evolution and mendelian genetics [54], and the synthesis of evolution and development in the field of evo-devo [55]. With the advent and commercialization of technologies like scRNA-seq, there is a broadened opportunity for new syntheses [56]. Rich and complex datasets are increasingly available from understudied branches on the tree of life, and comparisons between species will invariably invoke evolutionary questions. By integrating phylogenetic thinking across fields, we can start to answer these questions and raise new ones.
